# Scandinavian exceptionalism? Civic integration and labour market activation for newly arrived immigrants

**DOI:** 10.1186/s40878-016-0045-8

**Published:** 2017-03-01

**Authors:** Karen N. Breidahl

**Affiliations:** Centre for Comparative Welfare Studies, Department of Political Science, Aalborg University, Fibigerstræde 1, DK-9220 Aalborg Ø, Denmark

**Keywords:** Civic turn, Labour market policy, Scandinavian welfare states, Newly arrived immigrants

## Abstract

Since the late 1990s, a wide range of so-called new civic integration policies aimed at civilizing or disciplining newcomers have been introduced. Consequently, migration scholars have discussed whether a converging restrictive ‘civic turn’ has taken place in Western Europe or whether national models have been resilient: Based on an in-depth historical and comparative analysis of labour market activation policies targeting newly arrived immigrants in Sweden, Norway, and Denmark since the early 1990s, the article contributes to the overall question: *To what extent do the institutional pathways of the Scandinavian welfare states prevail when confronted with newcomers?* Activation policies targeting newly arrived immigrants exemplifies how the ambition of states to promote functional, individual autonomy is also an important, ongoing process in diverse policy areas of the welfare state and not restricted to early integration instruments.

While the Scandinavian welfare states differ on a number of counts with respect to immigration control, national integration philosophies and citizenship policies, the article outlines how activation policies aimed at newly arrived immigrants share several features. One of the key factors in this turn involves path dependency from, among others, a lengthy tradition for strong state involvement and norms about employment. Another factor in this turn involves transnational policy learning. On some points, national versions of these policies are also found due to country-specific citizenship traditions, integration philosophies and party political constellations.

## Introduction

‘New immigration’ waves gained hold in Western European countries some 50 years ago, and the continuous immigration of groups since World War II has increased the ethnic and religious diversity in these countries. Concerns for the economic and cultural incorporation of these new inhabitants (particularly those from non-Western countries) have accompanied this diversity (Ersanilli, 2012). Consequently, various integration policies towards immigrants have been adopted and adjusted over the course of several decades. Since the late 1990s in particular, a wide range of so-called new civic integration policies have been introduced, often referred to as policy requirements set up as a precondition for entry, permanent residency, and/or naturalization (i.e., obtaining citizenship) aimed at civilizing or disciplining newcomers and promoting functional, individual autonomy. The civic integration literature disagrees on whether Western European countries are seen as increasingly converging, whereby national models (multiculturalism versus assimilationism) no longer make sense (Joppke, 2007) or whether arguing for the resilience of national models and policy legacies from national integration philosophies and citizenship traditions (Goodman, 2014; Jacobs & Rea, 2007; Mouritsen, 2013). There has been little concern in this debate with how these policies play out in the geo-political contexts of the Scandinavian welfare states. On the one hand, Sweden, Norway, and Denmark are renowned for sharing manifold features in terms of how their respective welfare states are set up and in their respective histories of migration (Esping-Andersen, 1990; Sainsbury, 2012). On the other hand, they are also renowned for being very distinct when it comes to citizenship policies and national integration philosophies (Brochmann & Hagelund, 2012). Comparing Sweden, Norway and Denmark therefore allows us to shed light on whether some of the shared features of these welfare states are reflected in how the civic turn is manifested in each of these countries or whether other policy logics are prevalent. The article thereby contributes to the overall question: *To what extent do the institutional pathways of the Scandinavian welfare states prevail when confronted with newcomers?*

In order to shed light on this question this study focuses on activation policies directly targeting newly arrived immigrants enrolled in an introduction programme and provides a systematic, historical, and comparative analysis of the developing trajectories behind these policies in Sweden, Norway, and Denmark since the early 1990s until 2015.

The overall generic term ‘activation policies’ (e.g. active labour market policy, workfare, active line, welfare to work) refer to a shift from passive to active policies, a stronger emphasis on active citizenship and a citizen–welfare state relationship with greater emphasis on duties and obligations than on rights. This marked a new era in the welfare state in the 1990s, where the equal opportunity principle was replaced or at least supplemented by a new paradigm emphasizing active citizenship (Johansson & Hvinden, 2007). The article demonstrates how the strong emphasis on active citizenship is not only reflected in the general welfare state policies towards individual citizens. Hence, activation policies in recent decades targeting newcomers have become the main tools for promoting and fostering functional, individual autonomy among newly arrived immigrants and the principle of conditionality has been put forward by introducing a closer link between income maintenance schemes and employment-promoting measures. The shared turn towards activation in Scandinavian integration policies should not be interpreted as a retreat from national models towards a more singular focus on labour market functionality and economic instrumentalism (see Joppke, 2007, p. 14–19). Instead, we are dealing with a shared feature of the Scandinavian countries’ self-understanding and immigrant integration models, which reflect how civic integration manifests itself in these countries. Path-dependent policy traditions of these countries representing a shared duty-to-work tradition (expanded considerably in the early 1990s) and a lengthy tradition regarding state involvement are therefore important driving forces. However, the shared features of the three welfare states have also created favourable conditions for transnational policy learning. Finally, national versions of these policies are also found when it comes to how active participation in introduction programmes is linked to residency and citizenship requirements and whether workfare principles have been introduced owing to country-specific party political constellations, citizenship traditions and integration philosophies in how to accommodate cultural diversity.

The next section elaborates on the policy subarea of activation policies for newcomers and how it is part of a broader civic integration arsenal. It also presents the methodological approach and empirical material. Then follow a presentation and discussion of the theoretical framework. The fourth section contains the empirical analysis of the developing trajectories behind activation policies in the three countries and the fifth section a theoretical discussion of how to interpret the findings.

## Labour market activation as a part of a broader civic integration arsenal

Civic integration policies refer to a manifold group of policy areas, including citizenship tests, naturalization ceremonies, language and civic-orientation courses, and modules for role-playing social interaction (Goodman, 2014). The civic integration literature has mainly been concerned with policies that serve as gatekeepers for obtaining citizenship and permanent and long-term residence permits. However, it is a main point of this article, that civic integration is not restricted to the naturalization trajectory and early integration instruments: The ambition of states to promote functional, individual autonomy and to ensure that newcomers become ‘good citizens’ is also an important, ongoing process in diverse policy areas of the welfare state, including civic education, childcare services, and labour market activation. Labour market activation policies have been treated very superficially in the civic turn literature; however, at the conceptual as well as the theoretical level, this policy area can be seen as part of a broader civic integration arsenal as there is a clearly connection between the so-called ‘active turn’ emphasizing, on the one hand, active citizenship and a citizen–welfare state relationship with greater emphasis on duties and obligations than rights, and on the other hand ambitions regarding civic integration and promoting active and productive participation by immigrants and their commitment to liberal-democratic principles (Goodman, 2012, p. 659). Most notably, Christian Joppke has pointed out how these two tendencies (or phenomena) share an extensive focus on obligations and how both create illiberal temptations: ‘Civic integration is an instance, like eugenics and workfare policies, of illiberal social policy in a liberal state’ (Joppke, 2007, p. 14), and “civic integration” is the equivalent on the part of immigrants to the “workfare” policies that the general population is subjected to in the context of shrinking welfare states (for the latter, see Handler, 2004), seeking to make people both self-sufficient and autonomous by illiberal means’ (Joppke, 2007, p. 16; see also Goodman, 2014, p. 1). In these writing the ‘active turn’ is interpreted as an overall change and practice and as something tantamount to shrinking welfare states. However, the more general labour market literature has demonstrated how activation policies cross-nationally can differ considerably in terms of principles, implementation and policy instruments (e.g. Barbier, 2004).

This study refer to ‘activation policies’ in a rather wide sense; it basically means that the unemployed must participate in activation programmes in order to be eligible for unemployment benefits or social assistance. The developing trajectory of this policy area can be studied from many different angles including the content, volume, activation instruments etc. Because this study is mainly interested in to what extent the institutional pathways of the Scandinavian welfare states prevails when confronted with newcomers three dimensions are singled out (see below), see Hernes and Tronstad (2014) for an in-depth comparative analysis of other dimensions. When analysing the institutional pathways it is important to be aware that some labour market researchers have argued that the specific institutional and normative features of the Nordic countries are also reflected in their activation policies (Barbier, 2004; Jørgensen, 2008) while others have been more critical about these conclusions (e.g. Johansson & Hvinden, 2007, p. 53; Lødemel & Moreira, 2014; Jørgensen, 2008). However, these studies are concerned with activation policies targeting citizens in general and not how the active, duty-based approach interconnects with integration policies targeting newcomers (Breidahl, 2012). The three dimensions below are therefore all based on the premise that the manner in which the activation policies generally have taken place is not necessarily reflected in how policies for newcomers are manifested.

*1) Which principles are predominant in the activation approaches targeting newly arrived immigration in Denmark, Norway and Sweden?* This dimension focus on the prevalence of overall principles such as ‘public responsibility for welfare’ (degree of state involvement), ‘work for all’ (Kildal & Kuhnle, 2005), ‘relatively generous systems of benefits’ and how ‘good citizens’ are constructed.

*2) What are the relationship between rights and duties in the activation approaches targeting newly arrived immigration in Denmark, Norway and Sweden?* As opposed to the first dimension outlined above this dimension focus more specifically on the relationship between rights and duties. As mentioned in the former section different approaches to activation have evolved cross-nationally. In the empirical analysis I will mainly distinguish between two approaches: The ‘conditionality approach’ and the ‘work-first approach’. The former is characterized by a strong emphasis on the conditionality of welfare benefits, where the relationship between rights and duties is redefined by introducing a closer link between income maintenance schemes and employment-promoting measures (Johansson & Hvinden, 2007). By applying this approach, social benefits can basically remain generous. However, benefits are abolished or reduced in the event of non-attendance. The second activation approach implies a strengthening of the financial incentive to find employment by cutting income benefit levels and ‘making work pay’ as a point of departure. This approach is often referred to as work-first and usually related to strategies found in the Anglo-Saxon countries (Barbier, 2004). However, according to recent comparative research on activation policies we have increasingly to do with a more general approach in Europe and the US (Lødemel & Moreira, 2014). This approach differs from the conditionality approach in important ways, not least with respect to social citizenship, as the work-first approach more directly challenges universalistic principles and notions of social rights. Even though these two approaches above are presented as alternatives they can in practice co-exist side by side.

*3) How are activation linked to settlement and naturalization requirements in Denmark, Norway and Sweden?* Because we are interested in how labour market activation is part of a broader civic integration arsenal it is of course of crucial importance to identify how labour market activation is linked to settlement and naturalization requirements.

These three dimensions contributes to the overall question of to *what extent the institutional pathways of the Scandinavian welfare states prevail when confronted with newcomers* and the following section elaborates on how these dimensions are related to the theoretical framework. Theoretically are the historical institutional argument and the role of path dependency traditions brought to the fore. However, the ambition of the theoretical discussion is not sorely to test the explanatory power of the path dependency argument but more generally to understand the underlying driving forces. The theoretical framework will therefore also draw on theoretical insights from literature emphasising transnational learning mechanisms, citizenship traditions and national integration philosophies as well as the role of party, political constellations.

The comparative research design of the article allows an in-depth study of the character of the policies adapted over time together with their underlying driving forces. Hence, the empirical analysis will uncover the developing trajectories of the three dimensions of activation policies targeting newly arrived immigrants enrolled in an introduction programme and the receivers of a so-called ‘introduction allowance’ – specifically targeting newly arrived immigrants. This rather short but detailed examination is intended to take the complexity of the developing trajectories into account and is therefore based on a conjunctural, causation assumption, ‘[w]here those paths may be made up of combinations of more than one causal condition’ (Goodman, 2014, p. 67). The empirical analysis is furthermore based on the process tracing idea, where the underlying driving forces are identified by a thick description of how these policy changes came about. The empirical analysis of the policy development and the underlying driving forces are based on a comprehensive documentary body of material consisting of official documents published by the states, archival evidence, and secondary literature. Semi-structured interviews have also been conducted with a number of key figures (18 interviews) in the three countries, including civil servants, selected politicians and committee representatives, and working groups (for a thorough presentation of some of the empirical material, see Breidahl, 2012).

## Theoretical framework: the institutional pathways of the Scandinavian welfare states

The concept of a ‘Nordic (or Scandinavian) Model’ has been much disputed among social scientists and it has been questioned whether it still serve as an important reference category for comparative work (Kautto, 2010, p. 586–600). Furthermore, ‘… more recent comparative research and national studies have called into question the uniformity of the Nordic countries and their continued path dependency’ (Kautto, 2010, p. 600). When referring to the common institutional pathways of the Scandinavian welfare states this article does not arguing for Scandinavian unity or stability and it is important to acknowledge mutual national differences (e.g. in the concrete institutional frameworks). However, looking from the outside the three countries appear as fairly similar when it comes to their common (relatively) comprehensive and generous systems of benefits (which are largely universal), relatively similar labour market structures, and well-developed, family-friendly childcare services (Greve, 2007). Furthermore, it has been argued how crucial Nordic principles include ‘universalism’, ‘public responsibility for welfare’, and ‘work for all’ (Kildal & Kuhnle, 2005). Hence, researchers have pointed out how norms about employment have been stronger in Scandinavia than in most other countries (Svallfors, Halvorsen, & Andersen, 2001) and how the ‘Nordic countries have a long tradition of constructing the citizen primarily as a worker’ (Johansson & Hvinden, 2007, p. 56). The ‘work ethic’ has been attached to most of the adult population and it has been the dominant norm for many years that active participation in the labour market is of crucial importance for being accepted as an equal, full-fledged member of society. Finally, a lengthy tradition for strong state intervention has been prevalent and, compared to other countries, the state has played a crucial role in the implementation of activation policies in the Scandinavian countries (Jørgensen, 2008, p. 86).

Theoretically the historical institutional argument emphasise how institutions play a role in structuring behaviour and how path-dependent policy traditions as well as policy legacy from the past are of crucial importance in this process (Steinmo, 2008). More precisely these mechanisms refer to how the ‘…past influences present-day politics through a variety of mechanisms, ranging from concrete political institutions to patterns of interests associations to broadly accepted definitions of justice or even mundane ideas about the accepted way of doing things’ (Immergut, 2005, p. 289). Therefore, how policy processes unfold cannot solely be attributed to the presence of specific parties or the ideological orientations of governments based on the assumption that political events happen within a historical context where policy choices at one point in time affect choices at subsequent points in time (Pierson, 2000). Institutions structure and shape political behaviour and outcomes (Steinmo, 2008).

A unified theory of path dependency does not exist and the path dependency perspective can both figure as a rather narrow mechanism and as a broader approach. The last-mentioned is prevalent in this article. However, it is still important to develop criteria for when these mechanisms are present and when they are not. One important criterion, which I will focus on in the empirical analysis, is to what extent the institutional preconditions of the welfare state prevail when activation measures targeting newly arrived immigrants are introduced and, in continuation hereof, whether the three Scandinavian countries move in different directions and break with existing institutions and principles. Hence, based on these theoretical insights one might expect that the labour market activation policies targeting newly arrived immigrants in Denmark, Norway and Sweden, including the three dimensions outlined in the former section, would have similar features and over time move in the same direction due to processes of path dependency from these relatively similar features and principles. More precisely, one might also expect that the rather, active and paternalistic, duty-based approach targeting citizens in general that has come to dominate the welfare systems of all three countries in recent years are also manifested in policies towards newcomers due to institutional spill-over mechanisms from general changes of the welfare state. Finally, one might expect that the generous systems of benefits are maintained in the activation approaches targeting newcomers.

As mentioned above, the ambition of the article is not sorely to test the explanatory power of the path dependency argument but more generally to understand the underlying driving forces. This opens up for the relevance of applying alternative theoretical frameworks. One of these frameworks is transnational policy learning mechanisms. As historians have pointed out intra-Nordic transnational learning and lessons has been an important factor in the shaping of the national welfare systems (Kettunen & Petersen, 2011) and it raises the question of whether transnational policy learning mechanisms have been involved in the formation of this policy area from the 1990s and upwards. Research has shown how transnational learning mechanisms were present back in the 1960s and 1970s in the area of immigration and integration policy (Brochmann & Hagelund, 2012). The methodological approach based on process tracing and thick description allows us to take these dynamics into account by studying whether these mechanisms are prevalent. Furthermore, based on these theoretical assumptions one could expect to find common approaches to activation for newcomers in the three countries.

It is well-known how Sweden, Norway and Denmark differ on numerous points in their policy approaches adopted in response to immigration including the conditions for obtaining permanent residence and citizenship (Brochmann & Hagelund, 2012). Denmark has been labelled as the ‘outlier’ as regards the policy responses to immigration in the 1990s and 2000s and the implementation of restrictive citizenship policies. At the other end of the spectrum is Sweden, which has been idealized as a progressive country with respect to diversity, pluralism, and ethnic equality. Norway is somewhere in the middle (Brochmann & Hagelund, 2012). Based on these assumptions, one might expect that the turn towards activation has gone in different directions due to different integration philosophies and citizenship traditions and that these factors are more important than path dependent policy traditions and institutional spill-over mechanisms; especially when it comes to how labour market activation targeting newly arrived immigrants are linked to settlement and naturalization requirements.

From the literature it is also well-known how the party-political constellations of the three countries as well as immigration as a political issue differ considerable between the countries. Particularly Sweden and Denmark have for several years been known as complete opposite. Among others Green-Pedersen and Krogstrup (2008) have shown how the party political attention to this issue in the 1990s has been considerably stronger in Denmark than in Sweden as integration policy in Denmark has been much more conflict-laden than in Sweden. Based on these insights one could argue that a conflict-laden environment have an impact on the sub-area of activation policies for newly arrived immigrants. Consequently, one could anticipate that these features are more important than common features of the Scandinavian welfare states such as generous universal benefits: Especially when uncovering how the relationships between activation policies targeting newly arrived immigrants are related to the naturalization trajectory.

## Newly arrived immigrants and the Scandinavian version of active citizenship

Since the 1980s, when waves of immigration changed to consisting predominantly of refugees and family reunification, immigrants have been recognized as over-represented among those receiving social assistance in Denmark, Norway, and Sweden. It was first in the course of the 1990s, however, that the risk of welfare dependency became a main consideration as the employment gap between natives and immigrants increased considerably. Hence, compared to the native population (which has one of the world’s highest labor market participation rates), the employment rate among immigrants – men and women alike – has since remained relatively low (Breidahl, 2012), and the Scandinavian countries have been highlighted as facing great and extraordinary challenges in terms of integrating immigrants into the labor market due to the functional prerequisites of these comprehensive welfare states.

Over the years, reforms targeting newly arrived immigrants have been implemented and a new kind of social provision specifically targeting newly arrived immigrants has been introduced in the three countries in order to cope with this challenge. This benefit replaced the ordinary social assistance benefit in the introduction period – the so-called introduction benefit.^1^

### Denmark

The turn towards active citizenship in the area of integration policy in Denmark has been abrupt, prevalent, notable, and multi-pronged. Back in the 1970s and 1980s, however, Danish policymakers were very reluctant with respect to formulating immigration policy principles and a unifying national immigration policy. Moreover, when it came to introducing active employment-promoting measures targeting newly arrived immigrants, Danish policymakers were reluctant and (as in Norway at that time) voluntary organizations were responsible for taking care of newly arrived immigrants (Breidahl, 2012). In the mid-1990s, however, the political and administrative decision-makers became much concerned with integration issues; not least the low employment rates among immigrants, at the same time that the new paradigm of rights and duties was supplanting the equal opportunity principle in general welfare state policies.

In 1993, a committee was established to review the existing policy and formulate and submit new visions for this area. Consequently, the first national integration legislation was implemented under the Social Democratic government in 1999 (Law on integration of immigrants in Denmark (Act no. 474 of 1 July 1998); Breidahl, 2012, p. 62). In the area of labour market activation, this legislation included the introduction of an introductory programme and an introduction allowance targeting newly arrived immigrants. This programme implied strong conditionality, where a close link between income maintenance schemes and employment-promoting measures was introduced. Consequently, fines were imposed for non-compliance. The ordinary social assistance in Denmark was reformed in 1998, and the introduction programme for newly arrived persons followed more or less the same lines with respect to the strengthening of conditionality. Institutional spill-over mechanisms and timing were therefore key factors in understanding this conjunction of events – but only on some points. Hence, as opposed to the general reform of social assistance in 1998, the introduction allowance introduced in 1999 was considerably lower than the ordinary social assistance levels, whereby reduced benefit levels and the second activation approach – work-first – for the first time entered the area of integration policy (Andersen, 2007). The benefit levels were already returned to the level of ordinary social assistance in 2000, however, which was partially attributed to criticism from the UN Refugee Agency, UNHCR, which labelled the new introduction allowance as discriminatory (Breidahl, 2011). After returning to the level of ordinary social assistance in 2000, the introduction allowance was therefore (again) a copy of the reform of the ordinary social assistance in 1998, both regarding benefits levels and sanctioning principles.

After the November 2001 national election, immigration policy changed perceptibly when a Liberal–Conservative minority coalition government came to power with the support of the right-wing populist Danish People’s Party. One of the key issues – if not *the* key issue – for the latter was immigration and the perceived threat to Danish culture posed by immigration (Andersen, 2007).

Already in 2002, a so-called ‘start assistance’ or ‘introduction allowance’ (paid amount is the same) was introduced, which was to replace the former introduction allowance (equivalent to social assistance) for newly arrived immigrants. This new version of the introduction allowance was paid the first 3 years after a person has arrived in Denmark, and they obtained a resident permit if they participated in an introduction program. They then became eligible to receive ‘start assistance’ for a 4-year period. The benefit level was to remain the same for the entire 7-year period. As in 1999, these benefits were also considerably lower than the ordinary social assistance, but this time around the UNHCR did not raise the same criticism, as it was now also aimed at citizens with Danish roots who had returned after spending 7 years or more outside of Denmark. Start assistance/introduction allowance was some 35–50% lower than ordinary social assistance, depending on the family situation (the reductions being lower for families with children) (Andersen, 2007). While Danish social assistance was comparatively generous, the start assistance and introduction allowance schemes were among the *least* generous schemes in north-western Europe (Hansen, 2006). Like the first version of the introduction allowance in 1999, the principle of conditionality remained prevalent, meaning that the benefit was reduced correspondingly in the event of absence from the introduction programme (that could not be excused by illness or other compelling reasons). The formal political argument for introducing lower benefits was to reinforce the financial incentive to find employment and, as in 1999, there was greater emphasis on conditionality, meaning that absence from employment-promoting measures would reduce benefits correspondingly. The administrative framework for the introduction allowance (and start assistance) therefore differed little from the administrative framework of the Danish social assistance scheme (standardized rates for payment, where the exact rate depends on factors such as marital status, number of children, property, and the like, as opposed to the discretion of the municipal authority) and institutional spill-over mechanisms from general programmes for unemployed people to programmes for newly arrived immigrants was to some extent still apparent.

The September 2011 election resulted in a new coalition government consisting of the Social Democrats, Socialist People’s Party, and Social Liberals, and the political situation again changed dramatically in the immigration policy area. The start assistance and low introduction allowance were immediately abolished. Instead, the benefits for newly arrived immigrants were made comparable to ordinary social assistance throughout the introduction period. Removing these – by Danish standards – low benefits (so-called poverty benefits) was a main election issue for these three parties.

Four years later, however, in the summer of 2015, a right-wing government again came to power and promptly re-introduced a new version of the introduction benefit targeting newly arrived immigrants (came into effect on 1 September 2015). Like the start-assistance from 2002, the main ambition was to block the influx of refugees and promote labor market participation among refugees and immigrants; formally, it targeted anyone who has not lived in the Kingdom of Denmark (including Greenland and the Faroe Islands) for at least seven of the last 8 years. Since September 2015, instead of benefits levels comparable to ordinary social assistance, newly arrived immigrants have been entitled to much lower benefits – the same amount as the state education grant (depending on age, family situation etc.). Those who have passed the second level of Danish language training receive an additional monthly allowance of DKK 1500 (aprox. €195). At the same time, a ‘*genoptjeningsprincip*’ was introduced whereby it is not possible to receive children’s benefits in the first period. Finally, newly arrived immigrants are not allowed to earn extra income while receiving this benefit (Hernes & Tronstad, 2014). Consequently, the former institutional spill-over mechanism from social assistance to the introduction allowance was eliminated.

As mentioned earlier, Denmark, Norway, and Sweden differ remarkably when it comes to citizenship traditions and recent changes hereto, where Denmark has the most restrictive legislation. Already from the beginning in 1999, the first version of introductory programme was indirectly related to permanent residency and citizenship requirements in Denmark. Hence, passing a Danish language test is a prerequisite for both permanent resident status and Danish citizenship but active participation in introduction courses is not – unlike in Norway, where it is a prerequisite. Furthermore, permanent resident status can be granted earlier if the applicant has held paid employment for at least 3 years, and financial self-sufficiency is a prerequisite for obtaining Danish citizenship (Brochmann & Hagelund, 2012, p. 256; Hernes & Tronstad, 2014). In 1999, active citizenship and labour market activation policies were therefore directly linked to civic integration and the ambition of promoting active and productive participation by immigrants together with commitment to liberal-democratic principles.

### Norway

In the mid-1990s, the political and administrative decision-makers began recognizing that the existing social assistance system was unable to handle the over-representation of immigrants among social assistance recipients and the risk of welfare dependency. The Labour government of the day therefore granted these problems serious consideration (St. Meld, 1996–97, p. 17), and a new Introduction Act and a universal introduction allowance for newly arrived immigrants (lasts up to 2 years) were introduced in 2004: An introduction act which was not subject of political disagreement in the Norwegian parliament. In the same period, notions of active citizenship and the importance of being self-sufficient became important principles in the general welfare policies, including integration policy.

The ambition of the Introduction act was, firstly, to get immigrants out of the social assistance system and, more specifically, to stimulate and motivate the target group (newly arrived immigrants) to remain in the programme while simultaneously promoting the transition to active labour market participation (Ot. prp. 2002/03:28). In order to fulfil these ambitions, conditionality was put forward by introducing a closer link between income maintenance schemes and employment-promoting measures. The introduction allowance was set to be the equivalent of twice the basic amount from the National Insurance Scheme (for full participation in a program). The ‘work-first’ approach, which implies a reduced income benefit level in order to strengthen the economic incentive to find employment, was not applied. Rather, the introduction allowance was at least as generous as ordinary social benefits as newly arrived immigrants were hitherto entitled (before 2004), and in some cases (if both spouses participate in the programme) substantially higher (Hernes & Tronstad, 2014).

The introduction allowance was furthermore characterised as a fixed-rate benefit with standardized rates for payment with a character of a universal benefit, where the social assistance in Norway was (and remains) largely means-tested and the degree of local autonomy high. Furthermore, the introduction allowance was equal for everyone, regardless of their place of residence, and depended on participation in activities such as language training, courses about Norwegian society, and employment-promoting measures. In the event of absence unrelated to illness or other compelling reasons, the benefit was reduced correspondingly. The administrative framework related to the introduction allowance, therefore, fundamentally broke with the framework related to social assistance in general concerning local autonomy and local variations concerning the content, scope, and quality of activation policies that had previously applied to newly arrived immigrants. Unlike Denmark, institutional spill-over mechanisms were therefore not prevalent. Instead transnational policy learning mechanisms can be identified. Hence, as mentioned above, Denmark already introduced an introduction allowance with a strong emphasis on conditionality in 1999. When Norway was going to reform their introduction effort, according to their own statements, civil administrators and experts in the Introduction Act Commission were looking to their neighbouring countries to learn from their policy experiences; in particular, experiences from the reform in Denmark in 1999. According to a civil servant in the Norwegian Ministry of Labour and a member of the Introduction Law commission, the prevailing notion at the time was that the existing ordinary social assistance system was not sufficient and it was necessary to go a step further in order to handle the problems preventing immigrants from entering the labour market, which was why they were examining the (at the time) new Danish reform. Denmark was seen as an important source of inspiration in this area. This shift is interesting, as Sweden had been the primary source of inspiration in the 1970s and 1980s (Brochmann & Hagelund, 2012). However, when the notions of active citizenship were entering the arena and the Norwegian policymakers began recognizing that more drastic changes were necessary, their attention turned to Denmark (Breidahl, 2012, Chapter 11). Transnational policy learning mechanisms can therefore help explaining why the introduction programme and allowance introduced in Norway in 2004 in many ways follows the same line as the Danish variant with respect to the emphasis on conditionality.

As part of the introduction act, it was decided that active participation in parts of the introduction programme (paid by the authorities) was directly related to the conditions for obtaining permanent resident status and Norwegian citizenship; the active citizenship and civic requirements were thereby directly related – newcomers must participate in the process actively. Hence, as part of the introduction act in 2004, it was decided that a lack of participation in language training and instruction about Norwegian society (300 h^2^) will have negative consequences for both permanent resident status and Norwegian citizenship (Hernes & Tronstad, 2014; Valenta & Bunar, 2010, p. 473). Conversely, neither passing a test nor being financially self-sufficient (i.e., not depending on welfare benefits) are prerequisites for Norwegian citizenship (as in Denmark). In light of recent waves of refugees and asylum seekers from Syria and elsewhere (at the time of writing), however, recent changes have also been made in Norway, including revisions to the citizenship requirements (passing a test about Norwegian society and a minimum level of Norwegian for applicants between ages 18–67).

### Sweden

Already in 1993, the first version of an introduction allowance was launched in Sweden, which became the first country to implement such an allowance. Subsequently, the municipalities were encouraged (but not forced) to grant individuals participating in an introduction programme an introduction allowance rather than social assistance. At the time, the objective of the introduction allowance was to emphasize the special character of the allowance granted upon first arriving in Sweden. This version of the Swedish introduction programme was voluntary for the municipalities and newcomers alike, and the introduction allowance therefore merely provided an alternative to social assistance, it was not a replacement. The municipal authorities were also responsible for setting the introduction allowance and decide whether or not to punish a lack of active participation. Consequently, the implementation of the introduction allowance varied from municipality to municipality: In some, the amount was the same as regular social assistance, while in other municipalities it actually exceeded ordinary social assistance (Integrationsverket, 2007). Until 2010, the administrative framework for paying the introduction allowance towards newly arrived immigrants therefore did not stand out much from the regular social assistance system, and, until 2010, the rights for newcomers remained substantially more important than duties (Djuve & Kavli, 2007).

After a Liberal–Conservative government came to power in 2006, however, the question regarding the effort and income sources provided for newly arrived immigrants returned to the agenda. In 2007, a commission was set up with the remit to review and submit proposals concerning the responsibility for and design and financing of refugee reception and other initiatives for newly arrived refugees and others (and their relatives) requiring protection (SOU, 2008, p. 58). According to the commission chairman of the reform – the introduction allowance in particular – was shaped by experts who were puzzling over ‘policy failure’ in the past (Heclo, 1974), and it was again an important consideration that the existing ordinary social assistance system (which, before 2010, was reflected in the introduction programmes for newcomers) was not sufficient for handling the problems preventing newly arrived immigrants from entering the labour market. The reform as a whole was not subject to notable political disagreement (Breidahl, 2012, p. 264–266).

Later, in November 2009, the Swedish government presented a proposition (Prop, 2009/10, p. 60) based on many of the proposals the commission submitted in 2008, and a government reform entitled ‘Labour market introduction of newly arrived immigrants – individual responsibility with professional support’ was implemented in December 2010. In many ways, the introduction allowance launched as part of this reform follows some of the same lines and considerations as the Norwegian introduction allowance from 2004; it was important to consider how it could serve as an alternative to regular social assistance and how to settle on benefit levels that were neither too high nor too low compared to the general social assistance. As in Norway, there was talk of a universal, fixed-rate introduction allowance, where the social assistance in Sweden was (and remains) largely means-tested and the degree of local autonomy high (although less than in Norway). Therefore, the administrative framework related to the introduction allowance in Sweden fundamentally broke with the administrative framework related to social assistance in general concerning local autonomy and local variations concerning the content, scope, and quality of the activation policies that had previously applied to newly arrived immigrants.

Unlike Denmark, workfare principles and ‘make work pay’ principles was not in stake in the 2010 reform by cutting benefits. Instead the importance of conditionality (the first activation approach) was prominent.

As mentioned above, the citizenship policy of Sweden is very liberal, which is also evident regarding the relationship between participation in the introduction programme and the right to obtain residency or citizenship. Hence, participation in the introduction programme (directly or indirectly) is not related to the conditions for obtaining permanent resident status and Norwegian citizenship and nor are there any requirements about financial self-sufficiency or not having received social welfare benefits.

## Theoretical discussion

The comparative analysis above has demonstrated how a rather similar trend in activation policies for newly arrived immigrants in Sweden, Norway, and Denmark took place from the early 1990s until 2015. However, on some points differences and divergent trends were also identified. The findings therefore raise the question as to why the institutional preconditions of the welfare state occasionally prevail when new measures targeting newly arrived immigrants are introduced while at other times the three countries move in different directions and break with existing institutions and principles.

### Common trends and processes of path dependency

The similar trend in activation policies in the three countries reflects how notions of active citizenship came to dominate the welfare systems of the three countries during the 1990s and how these remarkable changes were also crucial for how the introduction programmes targeting newly arrived immigrants have been shaped and reformed. Consequently, newly arrived immigrants in all three countries have largely been subjected to activation policies and strict conditionality, and a requirement for receiving the introduction allowance is active participation in labour market training and/or language courses. This strong focus on labour market functionality and productive citizens in integration policy is hardly unique to the Scandinavian countries, as this can be found throughout Europe where different types of introduction programmes have also been introduced (Goodman, 2014, p. 1; Joppke, 2007, p. 14–18). However, the design of these programmes is unique and exceptional to the Scandinavian countries: It is not enough to prove sufficient language qualifications, pass tests, and demonstrate a commitment to liberal-democratic principles to be considered a ‘good’ citizen and a ‘full’ member of the society. Newcomers must consistently demonstrate that they are active citizens and that they are also in the *process* of becoming ‘productive’ citizens. The Scandinavian countries are therefore also actively involved in preparing activation programmes – as a right as well as a duty – whereby the state has been involved in preparing these people for the labour market. We are therefore dealing with developed, extensive, state-sponsored integration programmes of a magnitude that is unique within the European context and beyond and where the welfare state institutions are largely involved in the daily lives of newcomers (Valenta & Bunar, 2010). Several European countries have to some extent taken steps to institutionalize the idea that integration of immigrants is a ‘state affair’. However, these programmes are most comprehensive and widespread in the Scandinavian welfare states (Breidahl & Jønsson, 2014).

This tradition for strong state involvement also characterizes integration policy in general, where state involvement in the daily lives of the newcomers aimed at promoting active citizenship has increased over the past 20 years and now entails ‘active intervention in the private lives of refugees and immigrants by professionals within the Scandinavian welfare system seeking to shape these population groups socially, culturally, physically and psychologically according to Scandinavian norms’ (Olwig, 2011, p. 185). This ‘Scandinavian exceptionalism’ also reflects how high labour market participation among residents as well as newcomers has been perceived as a crucial requirement for financing generous welfare programmes and ambitious political aims.

These findings support the theoretical assumption that the adoption of introduction programmes for newly arrived immigrants and the activation strategies upon which they are built rest upon – and have been to some extent structured by – the path dependency traditions and policy legacy as well as spill-over mechanisms from policies targeted at the population in general. In particular the developing trajectories in Denmark in the late 1990s demonstrate how the policy legacy from the general institutional framework of the welfare state reveals a significant role for the adoption of the Danish version of the introduction programme and how timing was of crucial importance. As mentioned above, a reform emphasizing rights and duties and which strengthened the conditionality for the receivers of social assistance was adapted in Denmark 1998, and the introduction programme for newcomers introduced in 1999 more or less copied the principles behind this reform when it came to the activation approach of conditionality.^3^

It is also interesting to note how there in the outset were important differences in the respective institutional frameworks from which the introduction programmes originate, namely the social assistance schemes in the three countries. Therefore, the empirical analysis also opens up for the importance of the time sequence according to which Denmark adapted the programme and centralized activation principles in 1999, Norway following in 2004 and Sweden in 2010. This raises the question as to why Norway and Sweden have introduced introduction programmes that share many of the same features as the Danish programme with respect to strong conditionality. When delving deeper into the empirical material is was demonstrated how policy transfer and learning processes were also a stake and how the driving forces behind these changes and the convergent trend have been national as well as transnational policy-learning mechanisms (Breidahl, 2012; Heclo, 1974).

### National peculiarities, party political constellations, and integration philosophies

On some points, the empirical analysis also challenges the image of Scandinavian exceptionalism and common pathways, as national peculiarities and even points of divergence were identified: This became apparent in the examination of how participation in and the completion of introduction programmes are related to requirements for obtaining permanent residency and citizenship. The connection is absent in Sweden, and in Denmark the relationship is indirect (relevant language test must be passed) and financial self-sufficiency is a requirement for citizenship. In Norway, active participation in parts of the integration programme (language training and instruction about Norwegian society) is a direct prerequisite for obtaining resident status and citizenship (Hernes & Tronstad, 2014), and in the time of writing revisions to the citizenship requirements are about to be enacted. These national versions of this connection reflect how the policy dynamics affecting welfare state policies can be integrated with citizenship traditions in very different ways. ‘Today Sweden has one of the most liberal citizenship policies in Europe, while Denmark has one of the most restrictive. Norway occupies an intermediate position between its Scandinavian neighbours’ (Midtbøen, 2015, p. 1).

Also the implementation of work-first principles in Denmark towards newly arrived immigrants challenges the image of Scandinavian exceptionalism and common pathways. Even though there was strong emphasis on activation policies for weaker groups in the labour market in Denmark in the mid-1990s and extensive changes took place, benefits were not reduced in order to strengthen the financial incentive to work for unemployed people in general. The implementation of workfare principles in 2002 primarily targeted immigrants from non-Western countries. However, some of those who had been receiving social assistance for an extended period of time were also affected by strengthening the financial incentives to work in 2002 and 2005. One of the key factors to capture the cuts to benefits in Denmark in the 2002–2011 period and again in 2015 seems to be the influence of the country-specific party political constellations in this period, particularly the influence of right-wing populist parties and the extent to which the lack of financial incentives to work was recognized as a major barrier to integration within each country. In the second part of the 1990s, integration and immigration became important, salient political issues and objects of party competition in Denmark (Green-Pedersen & Krogstrup, 2008) reflected in the establishment of the Danish People’s Party in 1995. In general the political environment of immigration and integration issues has been much more conflict-laden in Denmark compared to Sweden and Norway.

As mentioned above, the November 2001 national election resulted in a Liberal–Conservative minority coalition government relying on the support of the right-wing populist Danish People’s Party. That political factors matter is further demonstrated by the fact that the change in government in 2011 resulted in the removal of the lower benefits for newly arrived immigrants – and their subsequent reintroduction in 2015. Hence, it has been in periods where the Danish People’s Party has had substantial influence on the government that welfare cuts and a work-first approach targeting immigrants have taken place.

However, we have to bear in mind that Denmark is not the only Scandinavian country with an influential right-wing populist party. Since 2013, the right-wing Populist Party *Fremskridtspartiet* has been part of the coalition government in Norway without lower benefits for immigrants having been introduced. This point indicates how the differences in the prevalent national integration philosophies probably also matter and are important to take into account when trying to understand why the workfare approach in Denmark, unlike Sweden and Norway, has repeatedly been ‘turned up’ for newly arrived immigrants since the late 1990s, thereby challenging universalistic principles and notions of social rights. Varying philosophies of integration in the three Scandinavian countries are dating back to the 1960s and 1970s. At that time, important principles were founded in Sweden: ‘Equality, freedom of choice and cooperation’ (Prop, 1975, p. 26). The policy path founded in Norway in the 1970s and 1980s was inspired in many ways by Swedish policy (Brochmann & Hagelund, 2012). In both countries, these principles were therefore relatively deep-rooted when active citizenship began affecting immigration policy in the 1990s. In Denmark, on the other hand, these principles were not present at that time. Hence, during the 1970s and 1980s, Danish policymakers were much more reluctant in the discussion of integration issues. Instead, the main focus was on immigration policy, as refugees were generally viewed as a temporary phenomenon and it was believed that most of them would eventually return to their country of origin (Breidahl, 2012, p. 108–109). Studies have also shown how the ‘equality’ principle and equal social rights in relation to immigrants and welfare benefits were already being debated – albeit to a limited extent – in the late 1970s, where the rights of guest workers to receive unemployment benefits were questioned if their language skills were not sufficient (Breidahl, 2012, Chapter 11).

## Conclusion

Since the late 1990s, so-called civic integration policies have been introduced in various Western countries aimed at civilizing or disciplining newcomers and promoting functional individual autonomy. These changes have raised numerous conceptual and empirical questions. In particular, it has been widely discussed whether a converging restrictive ‘civic turn’ has taken place in Western Europe or whether national models have been resilient. In order to contribute to this debate, this article has provided an in-depth comparative and historical analysis of labour market activation policies targeting newly arrived immigrants since the early 1990s until 2015 and thereby contributed to the question of: *To what extent do the institutional pathways of the Scandinavian welfare states prevail when confronted with newcomers?* In order to shed light on this rather broad question three dimensions were singled out focusing on the overall principles, the relation between rights and duties in the applied activation approaches and how activation is linked to settlement and naturalization requirements.

The empirical findings provide a rather complicated picture as both common but also diverging trends were identified. A general reorientation towards active citizenship targeting not only citizens but also newly arrived immigrants was identified. The numerous reforms targeting newly arrived immigrants resulted in an ‘active turn’ and a closer link between income maintenance and employment-promoting services. Conditionality was thereby strengthened remarkably in all three countries. Hence, the introduction programmes targeting newly arrived immigrants have been the particular object of reform since the early 1990s and have consequently become more similar during the 2000s.

A strong interconnection between activation policies and the civic turn seems to be exceptional to the Scandinavian welfare states and should not be interpreted as a retreat from national models towards a more singular focus on labour market functionality and economic instrumentalism (see Joppke, 2007, p. 14–19). Instead, we are dealing with a shared feature of the Scandinavian countries’ self-understanding and their respective immigrant integration models. These findings illustrate how path-dependency policy traditions emphasizing the influence of common institutional feature of the Scandinavian welfare states are an important theoretical frame of reference. Furthermore, it was illustrated how these policies have been closely related to and inseparable from more general welfare state changes. However, we should also be careful not putting too much emphasizes on the role of path dependency traditions when interpreting these developing trajectories. As pointed out earlier, researchers have questioned the uniformity of the Nordic countries and their continued path dependency and it was interesting to notice how transnational policy learning also was an important mechanism behind the diffusion of the conditionality approach. Finally, the empirical analysis also demonstrates divergent patterns on some points due to country-specific integration philosophies, citizenship traditions, and party political constellations. Hence, differences between the three countries were identified with respect to how participation in the introduction programmes has been related to requirements for obtaining a permanent residence permit and citizenship and whether work-first principles have been introduced (the third dimension). In general, Denmark stands out as the most restrictive country and breaks with the fundamental principles of income security and generous benefits that otherwise characterize the Scandinavian welfare states.

In conclusion, then, the institutional pathways of the Scandinavian welfare states have prevailed to some extent when confronted with newcomers in the area of labour market activation which demonstrates how theoretical notions about path dependency are also important in order to grasp how developing trajectories of how civic integration policies are taken place in subareas of the welfare state. However, the developing trajectories are far from unequivocal and this article has only studied one out of several policy areas where civic integration is an ongoing process.

What the future will bring is an open question. The recent flows of refugees have thrust new problems onto the political agenda in all three countries – and will probably lead to new solutions.

## Footnotes

1 The target group for the introductory programme is defined differently: In Sweden and Norway, the programme only applies to so-called ‘humanitarian immigrants’ (refugees who have received residence permits on humanitarian grounds), whereas the target group is broader in Denmark (also including some immigrants resulting from family reunification).

2 Increased to 600 h in 2011.

3 Some of the same dynamics can also be rediscovered in Norway where the setup of the introduction programme targeting newly arrived immigrants served as inspiration for the so-called ‘Kvalificeringsprogram’ targeting long tern recipients of social assistance.
